# Comparative Transcriptomes Analysis of Red- and White-Fleshed Apples in an F_1_ Population of *Malus sieversii f*. *niedzwetzkyana* Crossed with *M*. *domestica* ‘Fuji’

**DOI:** 10.1371/journal.pone.0133468

**Published:** 2015-07-24

**Authors:** Nan Wang, Yi Zheng, Naibin Duan, Zongying Zhang, Xiaohao Ji, Shenghui Jiang, Shasha Sun, Long Yang, Yang Bai, Zhangjun Fei, Xuesen Chen

**Affiliations:** 1 State Key Laboratory of Crop Biology, Shandong Agricultural University, Tai’an, Shandong, China; 2 College of Horticulture Sciences, Shandong Agricultural University, Tai’an, Shandong, China; 3 Boyce Thompson Institute for Plant Research, Cornell University, Ithaca, New York, United States of America; 4 Shandong Centre of Crop Germ-plasm Resources, Shandong Academy of Agricultural Sciences, Jinan, Shandong, China; 5 Tobacco Laboratory, Shandong Agricultural University, Tai’An, Shandong, China; Key Laboratory of Horticultural Plant Biology (MOE), CHINA

## Abstract

Transcriptome profiles of the red- and white-fleshed apples in an F_1 _segregating population of *Malus sieversii f*.*Niedzwetzkyana* and *M*.*domestica* ‘Fuji’ were generated using the next-generation high-throughput RNA sequencing (RNA-Seq) technology and compared. A total of 114 differentially expressed genes (DEGs) were obtained, of which 88 were up-regulated and 26 were down-regulated in red-fleshed apples. The 88 up-regulated genes were enriched with those related to flavonoid biosynthetic process and stress responses. Further analysis identified 22 genes associated with flavonoid biosynthetic process and 68 genes that may be related to stress responses. Furthermore, the expression of 20 up-regulated candidate genes (10 related to flavonoid biosynthesis, two encoding MYB transcription factors and eight related to stress responses) and 10 down-regulated genes were validated by quantitative real-time PCR. After exploring the possible regulatory network, we speculated that flavonoid metabolism might be involved in stress responses in red-fleshed apple. Our findings provide a theoretical basis for further enriching gene resources associated with flavonoid synthesis and stress responses of fruit trees and for breeding elite apples with high flavonoid content and/or increased stress tolerances.

## Introduction

Apple (*Malus domestica Borkh*.) is a fruit tree that is grown worldwide, because of its strong ecological adaptability, high nutritional value and good storage qualities of its fruit. In many countries, apple is the main fruit that is consumed, and its health properties have been strongly recommended[1,2]. Apple production is challenged by strong inbreeding problem narrowing the hereditary basis of the plants, thus resulted in fruit of poor nutritional quality and trees having declining stress tolerance[3]. Therefore, effective utilization of wild apple germplasm resources such as *M*.*sieversii* in distant hybridization will promote not only breeding elite apple varieties with distinguished fruit quality and stress tolerance, but expanding the genetic basis and diversity of cultivated apple as well.

*M*.*sieversii* and its red-fleshed variant (*M*.*sieversii f*.*niedzwetzkyana*) are both wild apple resources native to the mountains of Central Asia in southern Kazakhstan, eastern Uzbekistan, Kyrgyzstan, Tajikistan, Northern Afghanistan and Xinjiang, China. It has recently been shown that the *M*.*sieversii* is the primary ancestor of most cultivars of the domesticated apple (*M*.*domestica*)[4–7]. *M*.*sieversii* and *M*.*sieversii f*.*niedzwetzkyana* both have strong stress tolerance and are thought of as the most primitive species. The fruits of these trees have rich diversity in fruit morphology, phenol content, volatile component, sugar acid compositions and functional components of which polyphenols and calcium are about three times more abundant than in the cultivated apple ‘Starking’ variety[8]. Among the 177 kinds of aroma components that have been detected, 90 components such as the acetals and lactones are specific to *M*.*sieversii* and have great potential to be exploited further[8–10]. Unfortunately, *M*.*sieversii* resources are on the verge of extinction because of human interventions such as the reclamation of farmland. Clearly, the protection and utilization of wild apple resources are extremely urgent [11]. To do this effectively, a strong scientific basis and the creation of technical systems for the protection of *M*.*sieversii* germplasm resources are required and should include original habitat protection, *in vitro* organ preservation, and remote nursery building. To contribute to these efforts, we have investigated the genetic structure of an *M*.*sieversii* population [12,13], conducted *in vitro* tissue cryopreservation[14], and built a core resource of germplasm[15,16]. In addition, we used *M*.*sieversii f*.*niedzwetzkyana* germplasm that was protected in the Luntai National Fruit Germplasm Resources Garden (Xinjiang Academy of Agricultural Science) as a parent, and took the lead in building F_1_ hybrid segregation populations of *M*.*domestica* ‘Fuji’ and *M*.*sieversii f*.*niedzwetzkyana* crosses [17].

Wang et al.[18] found that the branches, leaves, flowers and fruits of *M*.*sieversii f*. *niedzwetzkyana* were all red, similar to the ‘Redfield’ variety. ‘Redfield’ is a type 1 red-fleshed apple of which the biosynthesis of anthocyanin was reported to be regulated by the MsMYB10 transcription factor[19]. This is different from the type 2 red-fleshed apple varieties, e.g.,‘Sangrado’ and ‘JPP35’, which have green foliage while red flesh in the fruit cortex [20,21]. Wang et al.[22] found that anthocyanin and flavonoid content, and antioxidant ability were much higher in ‘Zihong1’ red-fleshed strains. In addition, the culture system of red-fleshed apple callus had been established forvarious molecular mechanism studies of *M*.*sieversii f*. *niedzwetzkyana* [23]. The metabolism and mechanisms for fruit flesh coloration development are still largely unknown; in particular, the phenotypic differences and the different total phenol and flavonoid contents between red- and white-fleshed strains need further investigation.

RNA sequencing (RNA-Seq)has been applied widely in many fields, especially in plant functional genomics. RNA-Seq has been a powerful approach to study fruit development and quality, bud development and stress responses in fruit trees [24–27].The complete *M*.*domestica* genome was published in 2010 [4], which has provided a foundational resource for apple RNA-Seq studies. In the present study, we used an F_1_ hybrid population of *M*.*sieversii f*.*niedzwetzkyana* as the parental material. Using the principle of bulked segregant analysis (BSA) [28], we selected red- and white-fleshed fruit strains that displayed extremely different phenotype(fruit color) and built RNA pools of near-isogenic lines. This enabled us to perform comparative analysis of their transcriptional profiles and screen differentially expressed genes closely associated with the target phenotype. Our aim was to identify flesh color and stress related functional genes useful for the scientific protection and utilization of *M*.*sieversii* germplasm resources and the sustainable development of the apple fruit industry all over the world.

## Materials and Methods

### Plant material and RNA isolation

**Fig 1:**
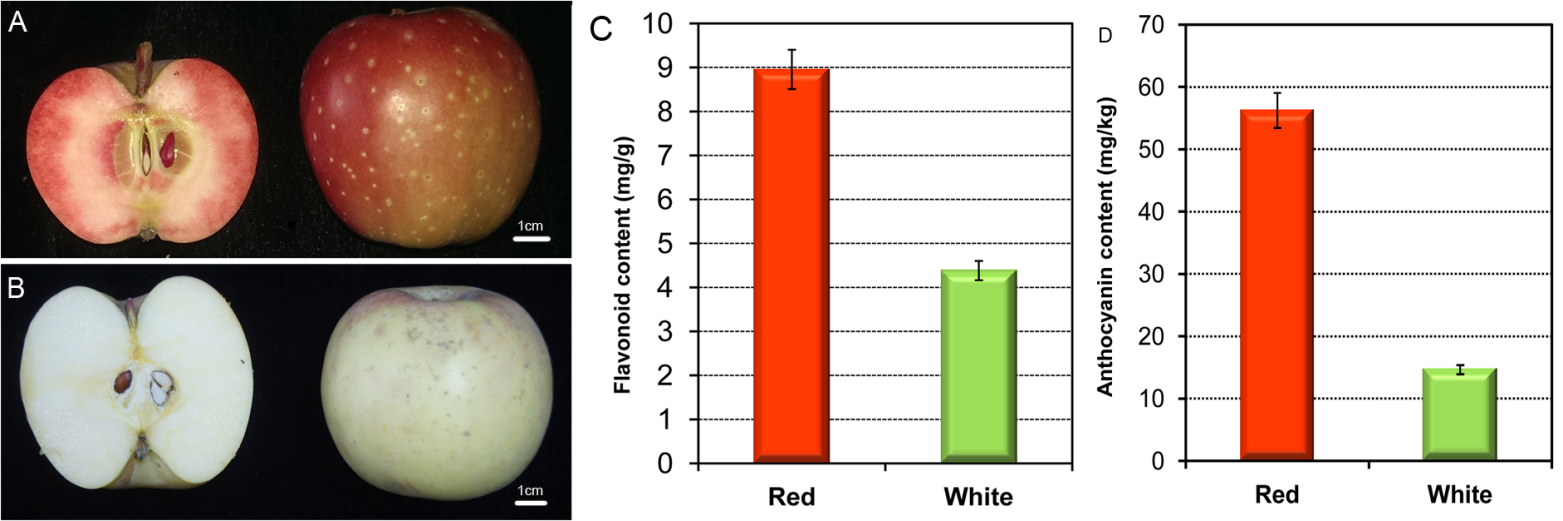
Red- and white-fleshed apples at the ripe stage used for RNA-Seq. (A)Red-fleshed apples in the F_1_ population. Scale bar = 1cm. (B) White-fleshed apples in the F_1_ population. Scale bar = 1cm. (C) Flavonoid content in red- and white-fleshed apples. (D) Anthocyanin content in red- and white-fleshed apples.

An F_1_ hybrid population derived from a cross between *M*.*sieversii f*. *niedzwetzkyana* and *M*.*domestica* ‘Fuji’ grown in the Shandong Agricultural University Tai’an Hengling Fruit tree breeding base(36°26′ N, 117°29′ E)was used in this study. Red- and white-fleshed fruits at the ripe developmental stage (Fig 1A and 1B) were harvested in biological triplicates, each from 20 F_1_ seedlings, then frozen in liquid nitrogen and stored at -80°C until use.

Total RNA was isolated using an RNAprep pure Plant Kit (Tiangen, Beijing, China)following the manufacturer’s protocol. The concentration (ng/uL) and quality (A260/A280) of the total RNA were determined using a Nanodrop 2000 spectrophotometer(ThermoScientific, USA), and the integrity of the RNA was tested on an Agilent Technologies 2100 Bioanalyzer. High quality total RNA in equal amount per sample was used to construct the RNA-Seq libraries, and a total of six libraries (three for the red-fleshed fruit and three for the white-fleshed fruit) were constructed.

Flavonoid content was determined using the method of Jia et al. [29]. Briefly, flesh samples were ground into powder in liquid nitrogen, then 1g of the powder was added to 10ml of 65% precooled ethanol for 4h at 4°C in dark. After centrifugation for 20 min at 12,000×*g*, 0.5 ml of the upper aqueous phase was removed and added to a test tube. Then 1ml of 5% NaNO2, 1ml of 10% Al(NO_3_)_3_, and 4ml of 2mol/l NaOH were added in order. After standing for 15min, spectrophotometric quantification was performed at 510nm using a UV–vis spectrophotometer (Shimadzu UV-2450, Kyoto, Japan). Rutin (Sigma Chemicals, Saint Louis, MI) was used as the master standard.

Anthocyanin content was measured using 0.5g of sample powder in 15ml of 1%(v/v) HCl-methanol for 24h at 4°C in dark. 1ml of the extracting solution was removed and added to two test tubes respectively,then 4ml KCL buffer (pH = 1.0) and NaAc (pH = 4.5) buffer were added, extraction for 15min at 4°C in dark. After centrifugation for 5 min at 8,000×*g*, the upper aqueous phase was subjected to spectrophotometric quantification at 510nm and 700nm using a UV-vis spectrophotometer (Shimadzu UV-2450). Anthocyanin content was calculated by pH differential method[30].

### RNA-Seq library preparation and sequencing

Magnetic beads with oligo(dT) were used to enrich the mRNAs, and then fragmentation buffer was added to fragment the mRNAs. The short mRNA fragments were used as templates and random hexamers were used to synthesize first-strand cDNA. Then double-stranded cDNA was synthesized by adding buffer solution, dNTPs and DNA polymeraseΙ. The double-stranded cDNAs were purified by AMPure XP beads according to the manufacturer’s instructions, then repaired at the tail ends, poly(A) added and enriched by PCR amplification. Finally, we tested the inserts sizes in the cDNA libraries on an Agilent 2100 Bioanalyzer. The library products were sequenced on aHiSeq 2000 system (Illumina, San Diego, CA).Raw RNA-Seq reads have been deposited in NCBI sequence read archive (SRA) under accession number SRP058589.

### RNA-Seq data analysis and differentially expressed gene identification

Raw RNA-Seq reads were processed using Trimmomatic[31] to remove adaptor and low quality sequences. Reads shorter than 40bp were discarded. RNA-Seq reads were then aligned to the ribosomal RNA database [32] using Bowtie[33] and the mappable reads were discarded. The resulting high-quality cleaned reads were aligned to the apple genome [4] using TopHat[34]. Following alignments, raw counts for each apple gene were derived and normalized to reads per kilobase of exon model per million mapped reads (RPKM).

To identify genes that were differentially expressed between the red- and white-fleshed apples, raw count data was fed to edgeR[35] and the resulting raw p-values of multiple tests were corrected using false discovery rate (FDR)[36].Genes with fold changes ≥ 2, adjusted p-values < 0.05, and the minimum expression level of the three biological replicates in the higher expressed group/the maximum expression level of the three biological replicates in lower expressed group> 1.3. Differentially expressed genes were classified into various functional categories based on the annotations of their Arabidopsis homologues and GO term enrichment analysis was performed using the Plant MetGenMAP system[37].

### Phylogenetic analysis of MYB transcription factors

A subset of 29 MYB transcription factors in the apple genome whose expression showed at least 1.5-fold difference between red- and white-fleshed apples (S1 Table)were used for phylogenetic analysis. The corresponding Arabidopsis orthologues of these apple MYB TFs were identified through BLAST searches against the TAIR10 Arabidopsis protein database [38]. Full length protein sequences were first aligned by Clustal W (opening = 10, extension = 0.2). Phylogenetic analyses were conducted by MEGA5.1 software [39] using 1000 bootstrap replicates.

### Real-time RT-PCR validation

To validate differentially expressed genes, quantitative real-time RT-PCR (qRT-PCR) was performed in triplicate using the same RNA samples as were used for the RNA-Seq library construction. First-strand cDNA was synthesized from 1ug of total RNA using RevertAidTM First Strand cDNA Synthesis Kit (Fermentas, Hanover, MD). The qRT-PCR reactions were conducted with 20-time diluted cDNAs as templates and MaximaTM SYBR Green/ROX qPCR Master Mix kit (Fermentas) on an iCycler iQ5 system (Bio-Rad, Hercules, CA). The MdAct gene served as an internal control and the relative quantification of specific mRNA levels was performed using the cycle threshold (Ct) 2^-ΔΔCt^ method (SoftwareIQ5 2.0) [40].The primers used for the semi-quantitative and qRT-PCR are listed in S2 Table.

## Results

### Summary and assessment of RNA-Seq data

In the F_1_ population of *M*.*sieversii f*.*niedzwetzkyana* x *M*.*domestica* ‘Fuji’, the red and white apples displayed significant phenotypic differences; in particular, the flavonoid content in red-fleshed apples was two times higher than in white-fleshed apples (Fig 1C) and the anthocyanin content was 5 times higher (Fig 1D). A total of 63,357,430 reads were obtained from six libraries (i.e., the libraries for the red-fleshed and white-fleshed apples at ripe stages and sampled in triplicate) (Table 1). After processing, the total number of cleaned reads per library ranged from 7.2 to 9.2 million. An average of 6,664,834 (81.8%) red-fleshed apple reads and 6,628,959 (84.6%) white-fleshed apple reads were mapped to the apple reference genome sequence. The RNA-Seq results for genes expressed in the red- and white-fleshed apples are shown in S3 Table.

**Table 1:** Summary of RNA-Seq data in the red- and white-fleshed apple libraries.

Sample	Total reads collected	Total clean reads	Total % of clean reads	Total mapped reads	Total % of mapped reads	rRNA	%rRNA
**R-rep** ^**a**^ **1**	12103831	9183948	75.88	7432923	80.93	2919883	24.12
**R-rep2**	10362434	7600066	73.34	6345588	83.49	2762368	26.66
**R-rep3**	9769645	7674307	78.55	6215992	81	2095338	21.45
**Mean**	10745303	8152773	75.87	6664834	81.75	2592530	24.13
**W-rep** ^**b**^ **1**	9512243	7237125	76.08	6128568	84.68	2275118	23.92
**W-rep2**	11020733	7706887	69.93	6518143	84.58	3313846	30.07
**W-rep3**	10588545	8550748	80.75	7240167	84.67	2037797	19.25
**Mean**	10373840	7831586	75.49	6628959	84.64	2542253	24.51

^a^Red-fleshed apple replicates

^b^White-fleshed apple replicates

To evaluate the genome-wide gene expression levels in each library, correlation coefficient values were calculated in a pair-wise manner using the RPKM data for all the genes in each library. The correlation analysis indicated that biological replicated libraries for each tissue type had highly consistent transcriptome profiles, and revealed that differences in the fruit flesh color at a certain developmental stage only marginally changed the transcriptome profiles of the genes (S4 Table).

### Changes in gene expression profiles between red- and white-fleshed apples

**Fig 2:**
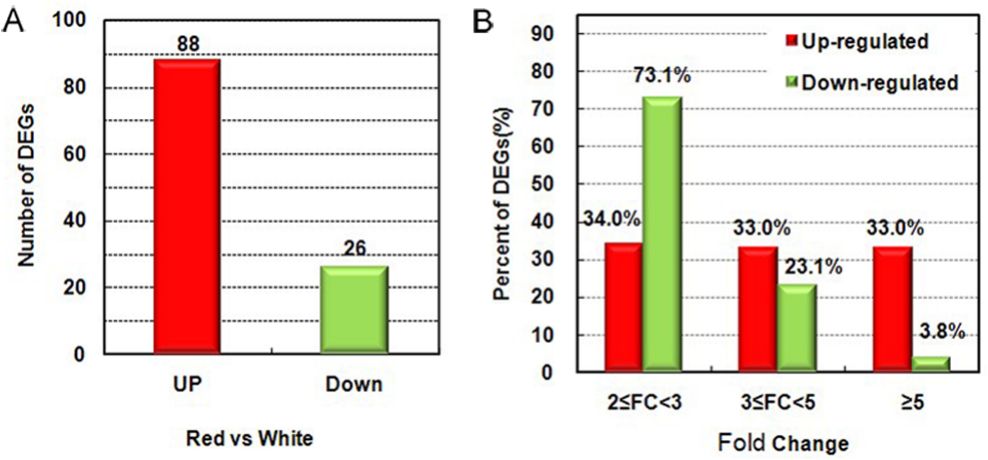
Analysis of differentially expressed genes (DEGs). (A) Numbers of up- and down-regulated genes between red- and white-fleshed fruits at the ripe stage. (B) Distribution of fold changes (FC) for up- and down-regulated genes at the ripe stage.

A total of 114 differentially expressed genes (DEGs)(S5 Table), including 88 up-regulated and 26 down-regulated, were identified in red-fleshed apples compared with white-fleshed apples (Fig 2A). The log_2_ratio of the gene expression ranged from -7.3 (down-regulated) to 8.4 (up-regulated).

As shown in Fig 2B, 96.2% of the down-regulated DEGs had fold changes in the 2–5 fold range, and only a small percentage of the down-regulated DEGs had fold changes≥5. Conversely, 33.0% of the up-regulated DEGs had fold changes ≥ 5, which implied that the up-regulated DEGs in red-fleshed apple may be more functionally significant.

**Fig 3:**
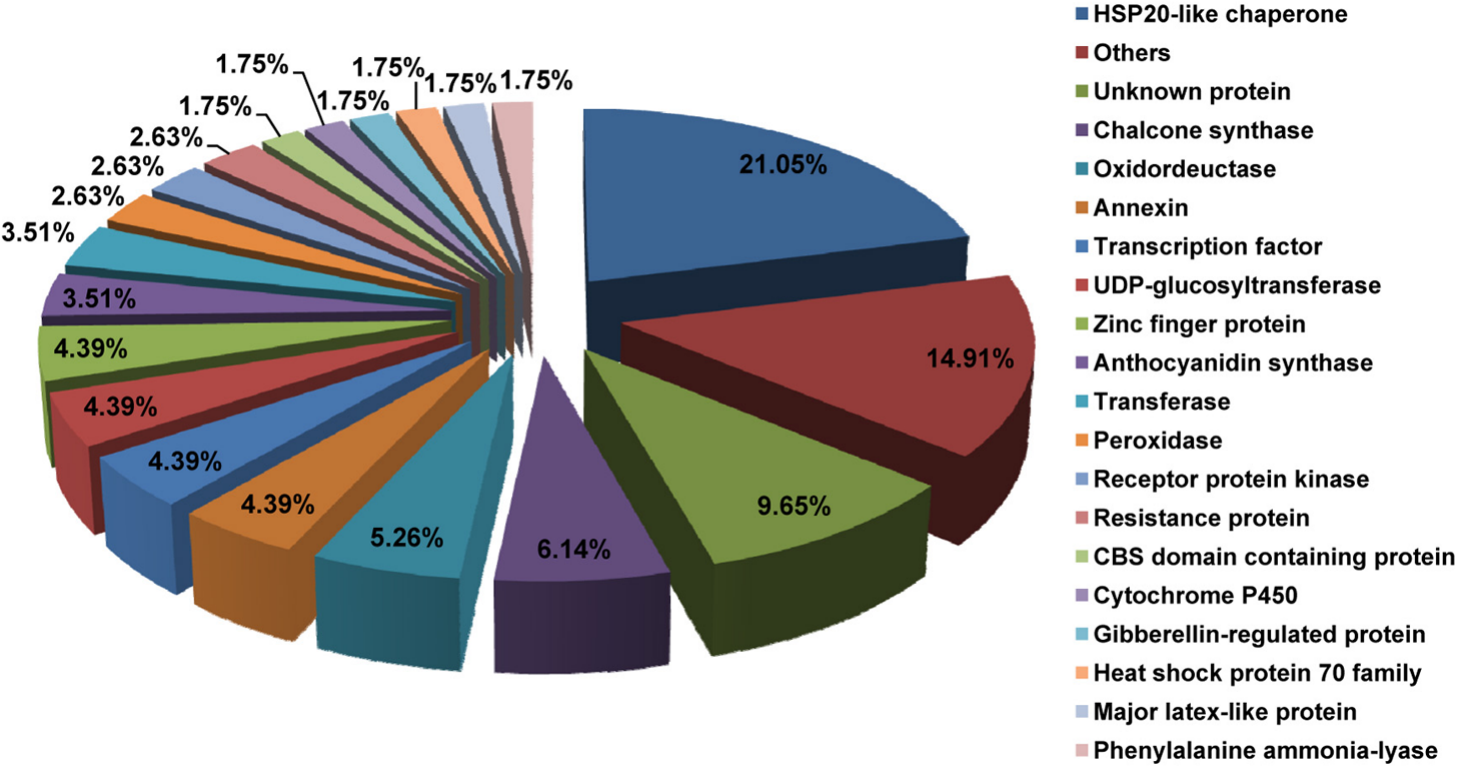
Functional categories of genes with changed expression between red- and white-fleshed apples.

To understand the functions of the DEGs, the genes were classified into 20 functional categories based on the annotations of the corresponding genes in the TAIR10 database. The largest category was HSP20-like chaperone (21.05%) (Fig 3), followed by unknown protein (9.65%), chalcone synthase (6.14%), oxidoreductase (5.26%), annexin (4.39%), transcription factor (4.39%), UDP-glucosyltransferase (4.39%), zinc finger protein (4.39%), anthocyanin synthase (3.51%), transferase (3.51%), peroxidase (2.63%), receptor protein kinase (2.63%), resistance protein (2.63%), CBS domain-containing protein (1.75%),cytochrome P450 (1.75%), gibberellin-regulated protein (1.75%),heat shock protein 70 family (1.75%), major latex-like protein (1.75%)and phenylalanine ammonia-lyase (1.75%).

### GO analysis of the DEGs between red- and white-fleshed apples

**Fig 4:**
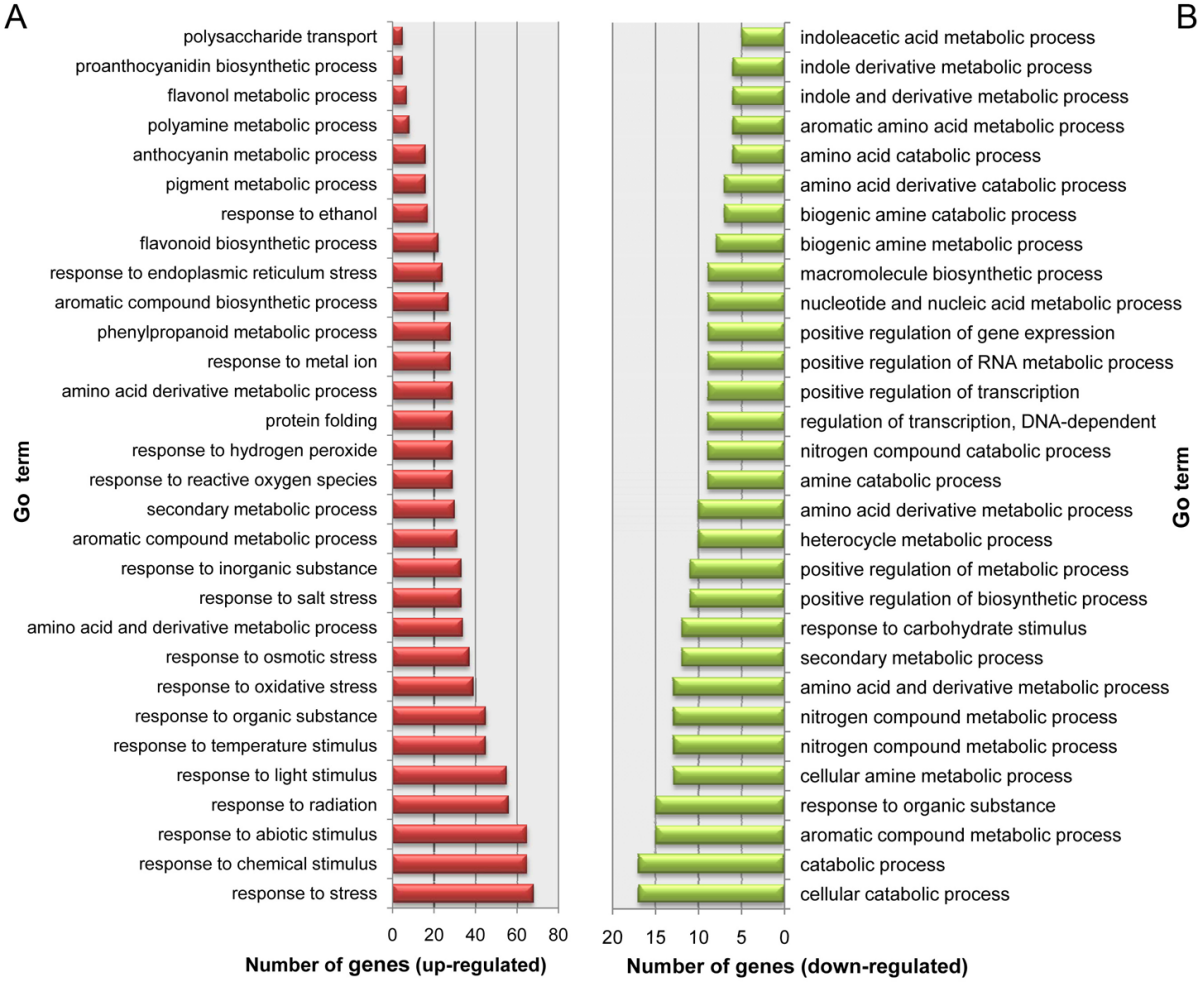
GO terms enriched in differentially expressed genes between red- and white-fleshed apples. (A) GO terms enriched in up-regulated genes in red-fleshed apple. (B) GO terms enriched in down-regulated genes in red-fleshed apple.

To gain insights into the functions of the DEGs, GO term enrichment analysis was performed on the DEGs. A total of 68 GO terms were enriched in the up-regulated genes and 32 GO terms were enriched in the down-regulated genes (S6 Table). The top 30 functional categories in the enrichment analysis are also shown in Fig 4. Among the 88 up-regulated DEGs in red-fleshed apples, 30 genes (34.1%) were associated with secondary metabolic process, 22 genes with flavonoid biosynthetic process, and 16 genes with anthocyanin biosynthesis(Fig 4A). Notably, GO terms associated with pigment metabolism, such as flavonoid and anthocyanin metabolic processes, were enriched in genes up-regulated in the red-fleshed fruits. Interestingly but unexpectedly, we found that 68 of the 88 up-regulated genes (77.3%) in the red-fleshed apples were related to response to different stresses, including chemical stimulus (65 genes, 73.9%), abiotic stimulus(63, 71.6%), light stimulus(55, 62.5%) and temperature stimulus (45, 51.1%). Seventeen of the 26 down-regulated genes (65.4%) in the red-fleshed apples, were related to catabolic processes, including nitrogen compound catabolic process (nine genes, 34.6%), biogenic amine catabolic process(seven genes, 26.9%) and amino acid catabolic process(six genes, 23.1%).

### DEGs related to flavonoids biosynthesis and metabolism

The 22 up-regulated genes in red-fleshed apples associated with flavonoid biosynthesis and metabolism processes are listed in Table 2. Some were well-known genes in the flavonoid synthesis pathway, including 4-coumaroyl:CoA ligase (4CL), chalcone synthase (CHS), chalcone isomerase (CHI), flavonoid 3′,5′-hydroxylase (F3',5'H), dihydroflavonol 4-reductase (DFR), anthocyanin synthase (ANS) and UDP-glucosyltransferase (UFGT). In addition, two of the genes were identified as MYB TFs (log_2_ ratio; red/white = 2.84 and 2.73) and one was identified as glutathione S-transferase (GST) (log_2_ratio; red/white = 2.89), indicating that they may play important roles in the development and regulation of red-fleshed apples.

**Table 2:** Up-regulated genes involved in flavonoid biosynthetic processes in red-fleshed apples.

GeneID	log_2_ ratio (red/white)	Description
MDP0000494976	1.56	Dihydroflavonol-4-reductase protein
MDP0000686666	2.54	Chalcone synthase; (Polyketide synthase, type III)
MDP0000134791	1.20	Chalcone isomerase
MDP0000788934	2.07	Anthocyanidin synthase;
MDP0000575740	2.28	Chalcone synthase; (Polyketide synthase, type III)
MDP0000175055	2.43	Multidrug resistance protein MdtK; (Multi antimicrobial extrusion protein)
MDP0000126567	2.53	Chalcone synthase; (Polyketide synthase, type III)
MDP0000360447	2.15	Anthocyanidin synthase protein; (Oxoglutarate/iron-dependent dioxygenase)
MDP0000259614	2.84	MYB transcription factor;(Homeodomain-like)
MDP0000127691	2.73	MYB transcription factor 10; (Homeodomain-like)
MDP0000190489	1.88	Cytochrome P450 flavonoid 3',5'-hydroxylase
MDP0000543445	2.35	UDP-glucosyltransferase, putative
MDP0000240643	2.04	Anthocyanidin synthase protein; (Oxoglutarate/iron-dependent dioxygenase)
MDP0000293578	2.30	4-coumarate: CoA ligase; (AMP-dependent synthetase/ligase)
MDP0000137655	2.70	Chalcone synthase; (Polyketide synthase, type III)
MDP0000478252	2.48	UDP-glucosyltransferase, putative
MDP0000405936	2.48	UDP-glucosyltransferase, putative
MDP0000252589	2.68	Chalconeflavonone isomerase
MDP0000686661	2.44	Chalcone synthase; (Polyketide synthase, type III)
MDP0000303216	2.44	WD-repeat protein, putative
MDP0000240641	2.14	Anthocyanidin synthase protein
MDP0000252292	2.89	GlutathioneS-transferase;(GlutathioneS-transferase,C-terminal-like)

### DEGs involved in response to stresses and related to the flavonoid content in red-fleshed apples

The GO enrichment analysis revealed that 68 up-regulated genes in red-fleshed apples were involved in response to stresses. Whereas among the down-regulated genes, some were found to be involved in response to organic substance and carbohydrate stimulus (Fig 4B). The up-regulated genes and their functional descriptions are listed in Table 3. Many of these genes were related to drought and cold tolerance, including heat shock protein (HSP), ascorbate peroxidase (APX), proline-rich protein (PRP), annexin (ANN), AN1-type zinc finger protein (ZFP), pyridoxal kinase (PLK) and the WRKY TF. These genes had significantly higher expression level in red-fleshed apples compared with white-fleshed, indicating that they may be crucial in the stress responses of red-fleshed apple. Further, the expression of genes encoding Dof zinc finger protein, aspartate aminotransferase, gibberellin-regulated protein and receptor-like protein kinase were down-regulated in red-fleshed apples.

**Table 3:** Up-regulated genes related to stress responses between red- and white-fleshed apples.

GeneID	log_2_(R/W)	Description	Possible function	Reference
MDP0000729108	2.86	Major latex-like protein	responses to various stresses	[42]
MDP0000261492	2.20	Phenylalanine ammonia-lyase	resistance to disease	[43]
MDP0000271528	2.17	CBS domain containing protein		
MDP0000632574	2.08	Major latex-like protein	responses to various stresses	[42]
MDP0000360447	2.15	Anthocyanidin synthase protein	responses to abiotic stress	[44,45]
MDP0000172108	2.27	17.5 kDa class I heat shock protein	tolerance to drought stress	[46]
MDP0000193724	3.32	Annexin	abiotic and biotic stress	[47,48]
MDP0000604702	1.14	17.5 kDa class II heat shock protein	tolerance to drought stress	[46]
MDP0000223568	1.67	18.2 kDa class I heat shock protein	tolerance to drought stress	[46]
MDP0000222593	3.19	Annexin	abiotic and biotic stress	[47,48]
MDP0000322202	2.63	Putative uncharacterized protein		
MDP0000935832	1.51	Heat shock 70 kDa protein		
MDP0000200564	2.27	Receptor protein kinase, putative; (Powdery mildew resistance protein, RPW8 domain)	Powdery mildew resistance	
MDP0000256650	1.38	Na+/Pi transporter; (Major facilitator superfamily)		
MDP0000175240	4.32	WRKY transcription factor, putative	stress response to cold and drought	[87]
MDP0000388415	4.22	Annexin	abiotic and biotic stress	[47,48]
MDP0000788934	2.07	Anthocyanidin synthase	responses to abiotic stress	[44,45]
MDP0000259614	2.84	MYB transcription factor	anthocyanin synthesis	[19]
MDP0000127691	2.73	MYB transcription factor	anthocyanin synthesis	[19]
MDP0000293578	2.30	4-coumarate: CoA ligase	phenylpropanoid metabolism	
MDP0000258055	2.13	NADH-ubiquinoneoxidoreductaserelated-like protein	oxidative stress	[49]
MDP0000523619	1.54	UDP-glycosyltransferase 1		
MDP0000252292	2.89	Glutathione S-transferase;	osmotic and salt stress	[50,51]
MDP0000208958	1.81	17.4 kDa class I heat shock protein 3; (HSP20-like chaperone)	tolerance to drought stress	[46]
MDP0000190489	1.88	Cytochrome P450 flavonoid 3',5'-hydroxylase	anthocyanidin synthase	
MDP0000520854	—^a^	Cinnamate-4-hydroxylase	anthocyanidin synthase	
MDP0000485762	4.54	blight-associated protein p12		
MDP0000255004	2.41	17.6 kDa class II heat shock protein	tolerance to drought stress	[46]
MDP0000240643	2.04	Anthocyanidin synthase protein	anthocyanidin synthase	
MDP0000259357	1.39	Disease resistance-like protein		
MDP0000151721	2.08	NADH dehydrogenase		[49]
MDP0000210077	1.19	Ascorbate peroxidase;	oxidative stress	[52]
MDP0000303216	2.44	WD-repeat protein, putative;		
MDP0000313080	2.19	Peroxidase;HSP20-like chaperone		
MDP0000320017	1.27	Xyloglucan endotransglucosylase		
MDP0000399965	1.22	Ascorbate peroxidase;	oxidative stress	[52]
MDP0000494976	1.56	Dihydroflavonol-4-reductase protein		
MDP0000414977	3.76	Annexin	abiotic and biotic stress	[47,48]
MDP0000154255	2.12	Pyridoxal kinase; Pyridoxal phosphate (active vitamin B6)	abiotic stress	[53]
MDP0000191304	2.23	Phenylalanine ammonia-lyase; (Aromatic amino acid lyase)	phenylpropanoid metabolism	
MDP0000211516	2.03	AN1-type zinc finger protein 2B	abiotic stress response	[54]
MDP0000240641	2.14	Anthocyanidin synthase protein	anthocyanidin synthase	
MDP0000638442	1.80	L-threonine 3-dehydrogenase		

^a^RPKM of MDP0000520854 in white-fleshed apples was zero.

Some of the up-regulated genes were found to be related to both stress responses and flavonoid biosynthesis (Table 4). The expression of genes encoding anthocyanin synthase in red-fleshed apples was much higher than that in white-fleshed apples, including the *M*.*domestica* genes MDP0000360447, MDP0000788934, MDP0000240643 and MDP0000240641. This suggested that anthocyanin was both associated with the red coloration and the stress tolerance of the red-fleshed apples. Furthermore, genes encoding MYB TFs, 4CL, GST, F3′5′H and WD-repeat protein also had higher expressional levels in the red-fleshed apples.

**Table 4:** Up-regulated genes involved in both the flavonoid biosynthetic process and stress response in red-fleshed apples.

Gene ID	RPKM (Red)	RPKM (White)	Arabidopsis ortholog	Gene symbol	Description
MDP0000360447	86.17	19.4	At4G22880	ANS	Anthocyanidin synthase protein;
MDP0000788934	77.74	18.57	At4G22880	ANS	Anthocyanidin synthase;
MDP0000259614	23.59	3.3	At1G66370	MYB10a	MYB transcription factor;
MDP0000127691	26.59	4	At1G66370	MYB10	MYB transcription factor 10;
MDP0000293578	48.66	9.9	At1G65060	4CL	4-coumarate: CoA ligase;
MDP0000252292	286.2	38.63	At5G17220	GST	Glutathione S-transferase;
MDP0000190489	63.11	17.13	At5G07990	CP450	Cytochrome P450 flavonoid 3',5'-hydroxylase
MDP0000240643	52.04	12.7	At4G22880	ANS	Anthocyanidin synthase protein;
MDP0000303216	3.74	0.69	At4G22880	WD40	WD-repeat protein;
MDP0000240641	90.48	20.57	At4G22880	ANS	Anthocyanidin synthase protein

### Phylogenetic analysis of MYB transcription factors in apple and *Arabidopsis*

**Fig 5:**
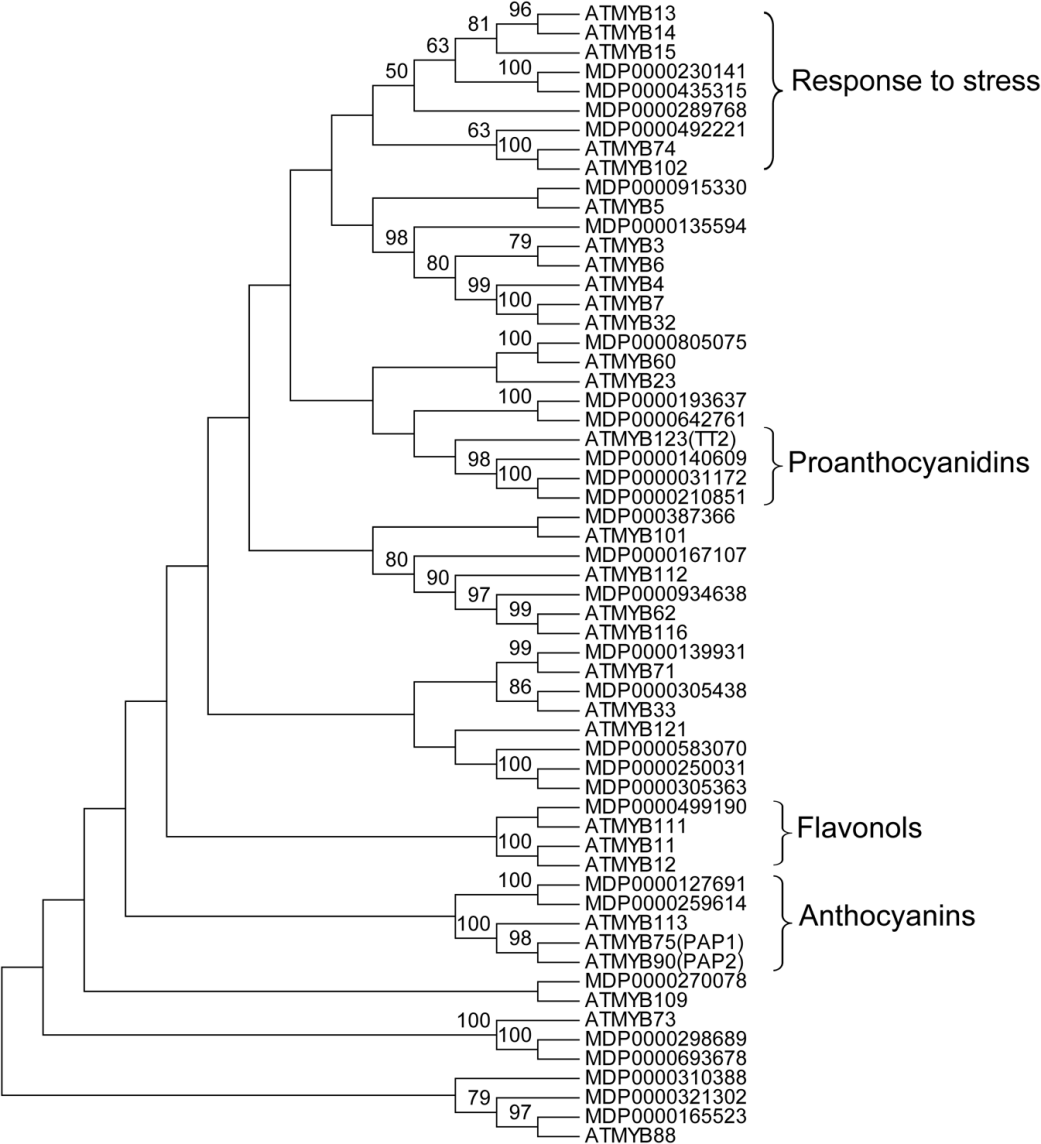
Phylogenetic analysis of MYB transcription factors in apple and their homologues in *Arabidopsis*.

MYB transcription factors (TFs) have been reported to play diverse functions in controlling pathways such as secondary metabolism, development, signal transduction, and disease resistance in plants [41]. A subset of apple MYB TFs and their corresponding Arabidopsis orthologues (see Materials and Methods)were used to construct a phylogenetic tree. The constructed phylogenetic trees showed that these MYB TFs formed several evolutionary branches (Fig 5), including groups associated with anthocyanin synthesis, proanthocyanin synthesis, flavonol synthesis, and stress response pathways. Some of the *Arabidopsis* MYB TFs have been identified as having various functions; for example, AtMYB12, AtMYB111 and AtMYB11 are involved in flavonol synthesis[55], AtMYB75 is involved in anthocyanin synthesis [56], while AtMYB15, AtMYB4, and AtMYB102 are involved in stress response pathways [57–59]. Comparative analyses of the apple and *Arabidopsis* MYB TFs that clustered on the same branches could provide valuable information about their functions.

### Verification of the expression patterns of DEGs related to flavonoid biosynthesis and stress responses

**Fig 6:**
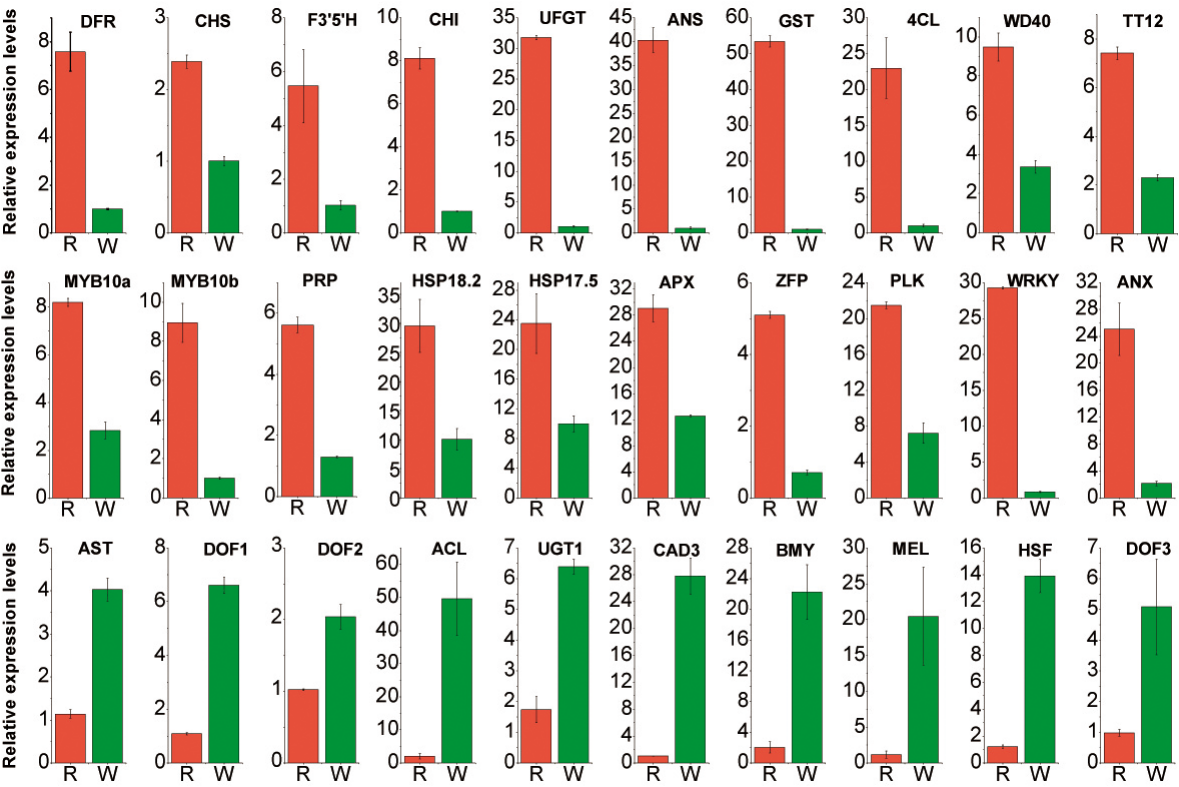
Verification of DEGs by qRT-PCR. The expression patterns of flavonoid synthesis-related genes in the red- and white-fleshed apples were validated by qRT-PCR. Actin was used as aninternal control.

To validate the gene expression results obtained from the RNA-Seq analysis, 30 DEGs (20 up-regulated and 10 down-regulated) were selected for qRT-PCR verification (Fig 6). The 20 up-regulated genes included ten related to flavonoid biosynthesis, two encoding MYB (MYB-related)TFs and eight encoding multiple stress responsive proteins. We found that the RPKM values of most of the 20 up-regulated genes were highly consistent with the expression levels obtained by qRT-PCR; the exceptions were MDP0000788934 (encoding ANS), MDP0000252292(encoding GST), MDP0000543445(encoding UFGT) and MDP0000293578(encoding 4CL) (S7 Table). The differential expression levels of these four genes were much higher in the qRT-PCR data. In addition, the expression levels of MDP0000788934, MDP0000252292, MDP0000388415, MDP0000543445, MDP0000175240 and MDP0000293578 was differed by more than 10 times between the red- and white-fleshed apples, indicating that these genes may play a decisive role in the phenotypic development of the red-fleshed apple. Among the 10 down-regulated genes, the RPKM values of MDP0000131249, MDP0000282334 and MDP0000248148 were nearly consistent with the qRT-PCR results, but the expression levels of the other genes were higher in the qRT-PCR results. Despite some quantitative differences in expression levels, the trends of the expression levels were similar in both the RNA-Seq and qRT-PCR data.

### Flavonoid metabolism might be associated with stress responses in red-fleshed apple

**Fig 7:**
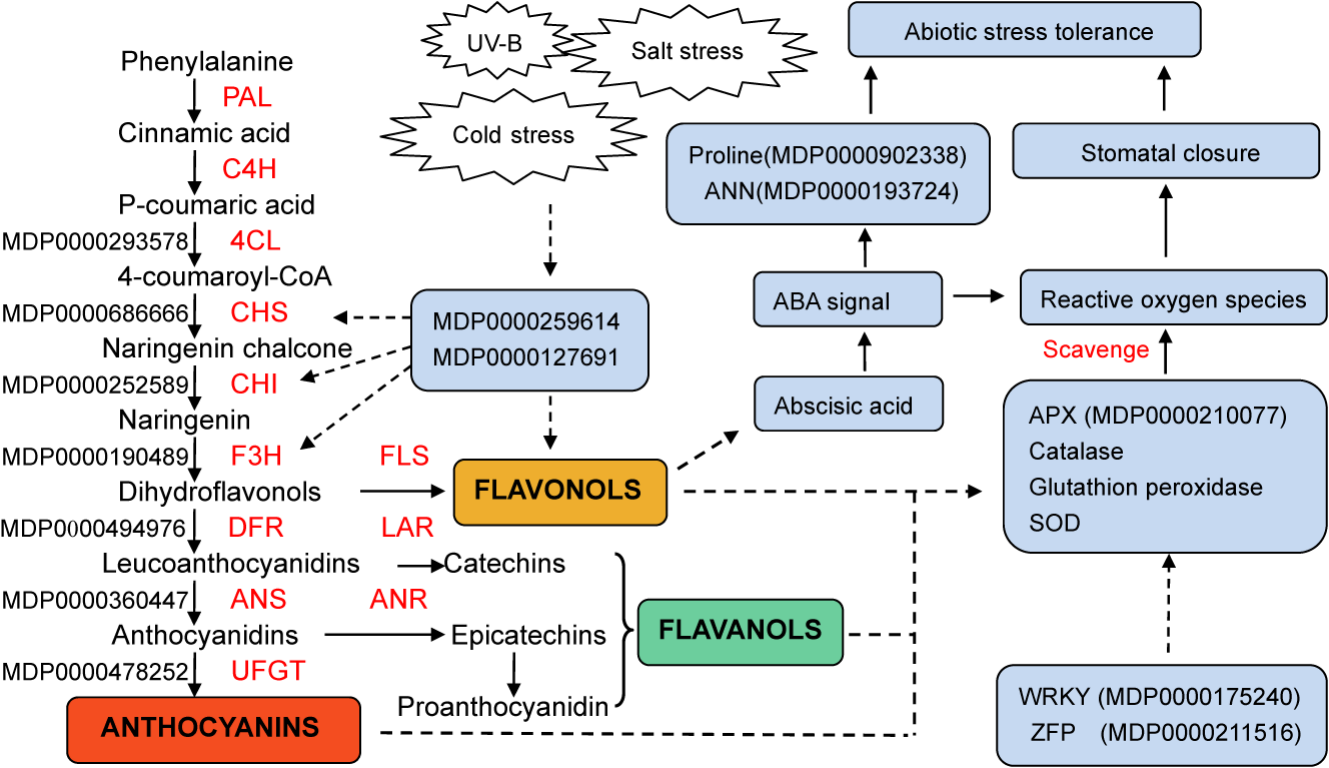
Model of the regulatory network involved in flavonoid metabolism and stress responses.

Stress tolerance is a multigene-controlled phenotype, which may include stress response, ion transport, secondary metabolism, and energyflow. The model that we constructed to illustrate our proposed regulatory network involving flavonoid metabolism and stress responses is shown in Fig 7. In other plants, it has been reported that UV-B radiation exposure, salts tress, drought stress and cold stress could induce plant cells to produce a large amount of reactive oxygen species (ROS),leading to oxidative damage to the cell. We found that the up-regulated genes in red-fleshed apples were associated with the accumulation of flavonols and flavanols. Studies have indicated that flavonoids might regulated the activity of ROS scavenging enzymes possibly involved in the stress response by scavenging ROS and regulating stomatal closure[60,61]. In this study, we also found that APX (MDP0000210077 and MDP0000399965) have significantly up-regulated expression in red-fleshed apples. Abscisic acid(ABA),an important plant regulator that is known to be involved in the stress response, was proved to be associated with flavonoid metabolism [62,63]. Flavonoids may increase the accumulation of ABA, and promote proline (MDP0000902338) [64] and annexin (MDP0000193724 and MDP0000388415) [65] genes that participate in stress responses mediated by ABA.

## Discussion

### Comparison of transcriptional regulation between red- and white-fleshed apples

Developmental mechanisms associated with red-fleshed apples have been studied widely [19–21]. According to Shu[66], red-fleshed apples were derived from Xinjiang wild apples into *M*. *sieversii f*.*niedzwetzkyana* and also from cultivated apples into *M*. *domestica varniedzwetzkyana*. However, Nocker et al. [5] identified 3,000 red-fleshed apple germplasm accessions in cultivated, wild and hybrid species drawing the conclusion that red-fleshed apples all originated from *M*. *sieversii f*.*niedzwetzkyana*. In 2006, our group developed F_1_ hybrid populations of red-fleshed apples by crossing cultivated apple *M*.*domestica* ‘Fuji’ with *M*.*sieversii f*.*niedzwetzkyana*. The red-fleshed apple parents we used were stored in the Luntai National Fruit Germplasm Resources Garden which also stores the oldest varieties of *M*. *sieversii f*.*niedzwetzkyana*. Chen et al.[67]reported that the phenotypic segregations exist in this F_1_ population of *M*. *sieversii f*.*niedzwetzkyana*, especially the red- and white-fleshed phenotypes. The anthocyanin content, flavonoids content, and antioxidant ability in red-fleshed F_1_ generation were significantly higher than in white-fleshed F_1_ generation, and higher than in white-fleshed ‘Golden Delicious’ and red-fleshed ‘Dehongcui’. The *M*.*sieversii* and *M*.*sieversii f*.*niedzwetzkyana*s to red in the Luntai National Fruit Germplasm Resources Garden were the most primitive species of red-fleshed apple and its F_1_ segregation population was distinctive and precious. Thus, the further exploration of these resources by RNA-Seq will help reveal the developmental mechanism of the red-fleshed phenotype. In this study, we investigated the transcriptional profiling of F_1_ population of *M*.*sieversii f*.*niedzwetzkyana* to explore transcriptional differences between red- and white-fleshed apples. The apple plants we used in this study were grown in the same environmental conditions and harvested randomly for pooling into three replicates per tissue type. For each tissue type, the mRNA library sequencing yielded 8.2 ± 0.9 million high quality reads per sample and 83 ± 2% of the clean reads were mapped to the apple genome. The correlation coefficient of gene expression between biological replicates ranged from 0.96 to 0.98, indicating good replicate consistency in this study (S4 Table). These results suggested that our RNA-Seq analysis was highly reliable.

A total of 114 genes were differentially expressed between red- and white-fleshed fruits. GO term enrichment analysis revealed that the up-regulated genes were significantly enriched with 68 biological processes and down-regulated genes with 32 biological processes(S6 Table).A high percentage of DEGs were associated with stress responses. In addition, flavonoid and anthocyanin metabolic processes were enriched in genes up-regulated in red-fleshed apples. Moreover, we suggested that the accumulation of flavonoid and anthocyaninin ripe apples may account for the red-fleshed phenotype strains in F_1_ populations of *M*.*sieversii f*. *niedzwetzkyana*. This accumulation was consistent with the high levels of flavonoid and high anthocyanin measured in red-fleshed apples [67]. However, in red-fleshed orange, the accumulation of lycopene was reported to be the cause of red-fleshed phenotype of ‘Hong Anliu’ [68].The enrichment of DEGs in red-fleshed apple related to response to different stresses was consistent with previous findings about stress tolerance in *M*.*sieversii* [69,70], indicating that the red-fleshed strains in F_1_ populations inherited the stress tolerance characteristics from *M*.*sieversii* and thus have more developed tolerance to stresses than white-fleshed strains.

Many studies have shown that the accumulation of anthocyanin pigments in plant tissues is a hallmark of plant stress. Anthocyanin plays essential roles in ameliorating environmental stresses induced by visible and UV-B radiation, drought and cold temperatures [71]. We also found that genes significantly differentially expressed were related to both stress responses and flavonoid metabolism (Table 4), confirming that anthocyanin did play an important role in plant stress. Flavonoids have been reported vital in response to stress in plants, such as protecting the plants from UV radiation, increasing tolerance of corn to aluminum toxicity and assisting the control of stomatal opening [72]. We also found that most DEGs involved in the regulation of flavonoids also were associated with stress responses (Table 4). Further studies are needed to investigate how flavonoids participate in stress responses in plants.

### Molecular processes and genes associated with the red-fleshed phenotype

High flavonoid content is an original feature of *M*.*sieversii f*. *niedzwetzkyana*[67]. As major polyphenol compounds of plant secondary metabolism, flavonoids are crucial not only in signaling between plants and microbial, but also in signaling between plant coloring matter and plant defensins. Furthermore flavonoids also have anti-bacterial, antioxidant properties as well as other health benefits for human [73–76]. Increases in the content of desirable components such as flavonoids are very important in apple breeding programs. In this study, among the 88 significantly up-regulated genes in red-fleshed apples, 22 were enriched in flavonoid biosynthetic process, including structural genes and TFs.

Enzymatic genes in the flavonoid biosynthesis pathway (e.g., PAL, CHS, CHI, ANS, UFGT and FLS) have been cloned in *Zea mays*, *Antirrhinum majus*, a Petunia hybrid, *Arabidopsis*, *Perillafrutescens* and other plants[77–80].In this study, eight genes encoding enzymes in the flavonoid synthesis pathway were significantly up-regulated in red-fleshed apples compared with white-fleshed apples, indicating that flavonoid synthesis in red-fleshed apples was significantly more activated than in white-fleshed apples. GST was reported previously to play a role in anthocyanin accumulation and transport [81]. Interestingly, we found that one GST encoding gene was expressed in a fruit color-dependent manner in red-fleshed apples, indicating GST might be involved in regulating fruit color in apple.

Members of the MYB TF family were reported as important regulators of fruit color. Stracke et al.[55] found that MYB11, MYB12 and MYB111 were highly correlated with flavonoid biosynthesis in *Arabidopsis*. In developing grape berries, Czemmel et al. [82] found that VvMYBF1 was a transcriptional regulator of flavonoid synthesis. The two genes (MDP0000259614, MDP0000127691) encoding MYB TFs we identified were significantly up-regulated in red-fleshed apples compared with white-fleshed apples. MDP0000259614 encoding MYB10 was reported previously to be a key regulator of apple fruit color[19]. In addition, the phylogenetic analysis between the MYB TFs from apple and *Arabidopsis*(Fig 5)showed that several apple MYB TFs were homologous to AtMYB75, involved in regulating the synthesis of anthocyanin, some were homologous to AtMYB12, AtMYB12 and AtMYB111, involved in regulating the synthesis of flavonols, and some were homologous to AtMYB15 and AtMYB102, involved in stress responses. These results indicated that apple MYB TFs may play crucial roles in flavonoid synthesis, fruit color regulation, and stress response in red-fleshed apple. Further studies of these MYBs and their interactions with each other will be important for exploring apple fruit color regulation.

Unexpectedly, we found that many of the DEGs between red- and white-fleshed apples were enriched for stress response terms. Further screening identified 8 genes related to various stress-tolerant pathways and the expression levels of these genes were validated by qRT-PCR. Genes encoding ANN, HSP, APX, PLK, WRKY TF and ZFP were significantly up-regulated in red-fleshed apples. Many of these genes were consistent with stress responsive genes reported in other plants, e.g., ANN was associated with the drought tolerance of *Brassica napus* L.var Q2 [83], sHSP was associated with the heat shock response of plants[84], APX played an important role in response to drought in African finger millet[85], ZFP245 improved the drought and cold tolerance by adjusting the proline content in rice[86]and WRKY38 participated in the response to cold and drought stresses in barley[87]. In apple, the WRKY TFs were identified as important regulatory factors in resistance mechanisms of apple ring rot, alternaria leaf spot, powdery mildew and abiotic stresses such as cold, high-salt and drought [88,89]. In this study, we found that a gene encoding WRKY was significantly up-regulated in red-fleshed apples; its expression was 20 times higher in red-fleshed apple compared with white-fleshed apple and the qRT-PCR result indicated that its expression level was 35 times higher in red-fleshed apple. The other stress responsive genes identified in the present study have rarely been studied in apple; however, their expression differences between red-and white-fleshed apples indicated that they were associated closely with stress response in red-fleshed strains. The extraction and use of these stress responsive genes in *M*.*sieversii f*.*niedzwetzkyana* may be important in apple resistance breeding. Furthermore, we tentatively explored the regulatory network between flavonoid metabolism and stress responses and proposed that it was not accidental that the up-regulated genes in red-fleshed apple were associated with both flavonoid metabolism and stress responses. The accumulation of anthocyanin, flavonols and/or flavanols could regulate stress responses by enhancing antioxidant activity and regulating stomatal closure and ABA metabolism. However, many unknowns still exist about the particular molecular regulatory mechanism between stress response and flavonoid metabolic pathways and further studies are needed.

## Conclusions

Here we report differences in gene expression between red- and white-fleshed fruits in an F_1_ hybrid population of *M*.*sieversii f*.*niedzwetzkyana* crossed with *M*.*domestica* ‘Fuji’. We analyzed the metabolic mechanisms behind the red-fleshed phenotype and identified differentially expressed genes that were related to flavonoid synthesis and stress responses. We also explored the possibility of a regulatory network between flavonoid metabolism and stress responses. Our findings provide a scientific basis for further studies on breeding for high-quality and stress tolerance in apple.

## Supporting Information

S1 Table MYB transcription factors selected for phylogenetic analysis. (XLS)

S2 Table Primer sequences used for the qRT-PCR validation of selected differentially expressed genes. (XLS)

S3 Table Genes expressed in ripe red- and white-fleshed apples. (XLSX)

S4 Table Correlation coefficient values between replicated samples in the two libraries (based on gene expression). (XLS)

S5 Table Differentially expressed genes between red- and white-fleshed apples. (XLSX)

S6 Table GO enrichment analysis of differentially expressed genes between red- and white-fleshed apples. (A) GO terms enriched in up-regulated genes in red-fleshed apples. (B) GO terms enriched in down-regulated genes in red-fleshed apples.
(XLS)

S7 Table RPKM values and qRT-PCR data of 30 candidate DEGs. (XLSX)
